# Diffusion tensor imaging with direct cytopathological validation: characterisation of decorin treatment in experimental juvenile communicating hydrocephalus

**DOI:** 10.1186/s12987-016-0033-2

**Published:** 2016-05-31

**Authors:** Anuriti Aojula, Hannah Botfield, James Patterson McAllister, Ana Maria Gonzalez, Osama Abdullah, Ann Logan, Alexandra Sinclair

**Affiliations:** Institute of Metabolism and Systems Research, University of Birmingham, Edgbaston, Birmingham, B15 2TT UK; Centre for Endocrinology, Diabetes and Metabolism, Birmingham Health Partners, Birmingham, B15 2TH UK; Department of Neurosurgery, Division of Pediatric Neurosurgery at the Washington University School of Medicine and the Saint Louis Children’s Hospital, St. Louis, MO 63110 USA; Department of Bioengineering, University of Utah, Salt Lake City, UT 84112 USA; Neurotrauma, College of Medicine and Dentistry, University of Birmingham, Edgbaston, Birmingham, B15 2TT UK; Department of Neurology, University Hospitals Birmingham NHS Foundation Trust, Birmingham, B15 2TH UK

**Keywords:** Hydrocephalus, DTI, Cytopathology, Decorin

## Abstract

**Background:**

In an effort to develop novel treatments for communicating hydrocephalus, we have shown previously that the transforming growth factor-β antagonist, decorin, inhibits subarachnoid fibrosis mediated ventriculomegaly; however decorin’s ability to prevent cerebral cytopathology in communicating hydrocephalus has not been fully examined. Furthermore, the capacity for diffusion tensor imaging to act as a proxy measure of cerebral pathology in multiple sclerosis and spinal cord injury has recently been demonstrated. However, the use of diffusion tensor imaging to investigate cytopathological changes in communicating hydrocephalus is yet to occur. Hence, this study aimed to determine whether decorin treatment influences alterations in diffusion tensor imaging parameters and cytopathology in experimental communicating hydrocephalus. Moreover, the study also explored whether diffusion tensor imaging parameters correlate with cellular pathology in communicating hydrocephalus.

**Methods:**

Accordingly, communicating hydrocephalus was induced by injecting kaolin into the basal cisterns in 3-week old rats followed immediately by 14 days of continuous intraventricular delivery of either human recombinant decorin (n = 5) or vehicle (n = 6). Four rats remained as intact controls and a further four rats served as kaolin only controls. At 14-days post-kaolin, just prior to sacrifice, routine magnetic resonance imaging and magnetic resonance diffusion tensor imaging was conducted and the mean diffusivity, fractional anisotropy, radial and axial diffusivity of seven cerebral regions were assessed by voxel-based analysis in the corpus callosum, periventricular white matter, caudal internal capsule, CA1 hippocampus, and outer and inner parietal cortex. Myelin integrity, gliosis and aquaporin-4 levels were evaluated by post-mortem immunohistochemistry in the CA3 hippocampus and in the caudal brain of the same cerebral structures analysed by diffusion tensor imaging.

**Results:**

Decorin significantly decreased myelin damage in the caudal internal capsule and prevented caudal periventricular white matter oedema and astrogliosis. Furthermore, decorin treatment prevented the increase in caudal periventricular white matter mean diffusivity (p = 0.032) as well as caudal corpus callosum axial diffusivity (p = 0.004) and radial diffusivity (p = 0.034). Furthermore, diffusion tensor imaging parameters correlated primarily with periventricular white matter astrocyte and aquaporin-4 levels.

**Conclusions:**

Overall, these findings suggest that decorin has the therapeutic potential to reduce white matter cytopathology in hydrocephalus. Moreover, diffusion tensor imaging is a useful tool to provide surrogate measures of periventricular white matter pathology in communicating hydrocephalus.

**Electronic supplementary material:**

The online version of this article (doi:10.1186/s12987-016-0033-2) contains supplementary material, which is available to authorized users.

## Background

Hydrocephalus is a common paediatric neurosurgical presentation with an incidence of 0.48–0.81 per 1000 live births [1–3]. Communicating hydrocephalus is aetiologically heterogeneous; bacterial meningitis, subarachnoid haemorrhage, trauma, intracranial and intraspinal tumours as well as leptomeningeal metastases can all cause the disorder [4–10]. The incidence of communicating hydrocephalus following subarachnoid haemorrhage is at least 13 % and can be as high as 67 % [11]. In addition to ventriculomegaly, communicating hydrocephalus is accompanied by extensive global cerebral pathology, including widespread reactive gliosis, hydrocephalic oedema and demyelination [10, 12].

Although shunting is the current standard of care for children with hydrocephalus, the procedure is associated with severe complications that contribute to an increased patient morbidity [13–16]. Furthermore, academic attainment and social integration difficulties continue into adulthood for those with the disease [17–19]. Therefore, the development of novel therapeutic strategies to prevent the development of hydrocephalus or promote recovery is of critical importance. Our recent study (Additional file 1: Figure S1) supports the key role of transforming growth factor-beta (TGF-β) in communicating hydrocephalus, as decorin, a TGF-β antagonist [20–23] ameliorated subarachnoid fibrosis and therefore significantly attenuated the enlargement of the ventricular system [12]. However, the effectiveness of decorin to prevent cytopathology in hydrocephalus is yet to be examined thoroughly. Given that cellular pathology is largely responsible for the array of functional deficits observed clinically and contributes to the impairment in patient health-related quality of life, it is important to understand whether decorin can attenuate these alterations in vivo [10, 24, 25].

Greater insight into the cytopathological changes occurring in communicating hydrocephalus can be achieved with the use of advanced non-invasive magnetic resonance diffusion tensor imaging (DTI) [26]. DTI is a specialised magnetic resonance imaging (MRI) technique that examines tissue anisotropic properties and cerebral microstructural integrity [27, 28]. DTI yields a set of quantitative metrics, reflecting the magnitude along the principal axes of water diffusion, which are sensitive to changes in the underlying brain microstructure. Commonly used scalar DTI parameters such as axial (AD), radial (RD), and mean diffusivities (MD) (equivalent to the speed of motion in the principal axes of diffusion) or the fractional anisotropy (FA) (equivalent to a normalized aspect ratio of the principal axes of diffusion) have been useful in the investigation of cerebral abnormalities; an increase in the AD, RD and MD alongside a decrease in the FA occurs in the cerebral white matter of children with hydrocephalus [29–33]. Furthermore, the specificity of DTI to act as a surrogate measure of cerebral pathology has been highlighted in a variety of conditions, including hypoxic ischaemic injury [34, 35], multiple sclerosis [36–39], spinal cord injury [40], obstructive hydrocephalus [41], temporal lobe epilepsy [42, 43] and for delineating gliomas [44]. However, correlations between DTI parameters and underlying cytopathology in communicating hydrocephalus have yet to be determined (Appendix 1).

Therefore, using immunohistochemistry and clinically relevant neuroimaging we investigated whether decorin is able to attenuate damage-related parameters and if cellular changes in communicating hydrocephalus can be quantitatively characterised by DTI using a juvenile rat model of the disorder.

## Methods

### Experimental animals

Three-week-old Sprague–Dawley rats (Charles River, Massachusetts, USA) were housed in litters in individual cages, kept under a 12 h light/dark cycle with free access to food and water. Animals were monitored for adverse effects of treatments, such as distress, lethargy, weight loss and seizures, and any animals showing severe adverse effects were euthanised. Experiments were conducted at the University of Utah in accordance with the guidelines of the National Institutes of Health Care and Use of Laboratory Animals and approved by the University of Utah Ethics Committee.

### Experimental design and surgical techniques

The experimental design and surgical techniques are described in detail elsewhere [12]. Using a ventral approach, the interval between the occipital bone and the C-1 vertebral body was exposed and a 30 gauge angled needle was inserted into the prepontine (basal cistern) subarachnoid space. 30 µl of 20 % kaolin solution (200 mg/ml in 0.9 % sterile saline; Fisher Scientific, Massachusetts, USA) was injected to induce communicating hydrocephalus and the rat was either allowed to recover or underwent osmotic pump and intraventricular cannula implantations. The cannulae were inserted into the right lateral ventricle and fixed in place with glue and bone cement (Biomet UK Ltd, Bridgend, UK) to a stabilising screw, and connected to subcutaneously implanted mini osmotic pumps. Osmotic pumps (model 2002 adapted for use in MRI scanners with PEEK tubing, Alzet, Durect Corporation, California, USA) were filled with either 5 mg/ml human recombinant decorin (Galacorin^TM^, Catalent/Pharma Solutions, New Jersey, USA) or 10 mM phosphate buffered saline (PBS) pH 7.4 (Sigma-Aldrich, Missouri, USA). Over the subsequent 14 days, human recombinant decorin was infused at a rate of 2.5 mg/0.5 ml/h.

Rats were randomly assigned to four groups: (1) Intact age-matched controls (Intact group, n = 4); (2) basal cistern kaolin injections only (kaolin group, n = 4); (3) kaolin injection with intraventricular infusion of PBS (kaolin + PBS group; n = 6); and (4) kaolin injection with intraventricular infusion of decorin (kaolin + decorin group; n = 5). Magnetic resonance imaging (MRI) and diffusion tensor imaging (DTI) were conducted after 14 days of treatment to assess the extent of hydrocephalus before sacrifice, then the brains were removed and processed for histology.

### Magnetic resonance imaging and diffusion tensor imaging

Imaging experiments were conducted 14 days post injury using a 7-Tesla horizontal-bore Bruker Biospec MRI scanner (Bruker Biospin, Ettlingen, MA, USA) interfaced with a 12-cm actively shielded gradient insert capable of producing magnetic field gradient up to 600 mT/m. Animals were anesthetised using 1–3 % Isoflurane and 0.8 L/min O_2_ and their vital signs (respiration, temperature, heart rate and oxygen saturation percentage) were continuously monitored using a MR-compatible physiological monitoring system (SA Instruments, Stony Brook, NY, USA). Animals were placed in a 72-mm volume coil for signal transmission, and a quadrature surface coil was placed on the head for signal reception. Acquisition of T_2_-weighted MRI scans and ventricular volume analysis has been described previously [12]. DTI scans were conducted using spin echo diffusion-weighted sequences with single-shot EPI readout, with the following parameters (TR of 3760 ms, TE of 44 ms, 15 coronal 1 mm-thick slices, a field of view of 2.5 × 2.5 cm, and an in-plane resolution of 195 × 195 µm). Thirty uniformly-spaced over unit sphere diffusion-weighted gradient directions and five non-weighted images were acquired with two signal averages and the following diffusion parameters: diffusion gradient duration 7 ms, separation 20 ms, diffusion encoding sensitivity 700 s/mm^2^. Scan time was 4 min. For ventriculomegaly analysis, one MRI scan image was chosen from the rostral cerebrum (−0.36 mm from bregma) and the caudal cerebrum (−3.72 mm from bregma) for each rat, and the ventricular area was determined in each scan using ImageJ.

### DTI voxel based analysis

**Fig. 1:**
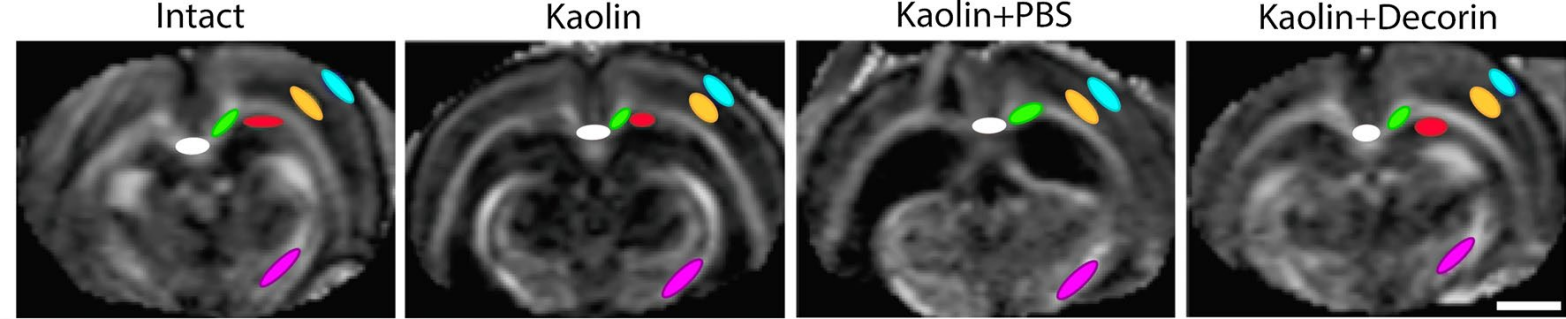
Regions of interest (ROIs) for DTI analysis in each experimental group. Representative voxel based map images and analogous diffusion tensor images of the caudal cerebrum at 2.76 mm posterior to Bregma are shown for the four experimental groups. All ROIs selected for analysis, except from the rostral internal capsule, are displayed. ROIs were chosen with the aid of a rat brain atlas [45]; *white* = corpus callosum, *green* = periventricular white matter, *cyan* = outer parietal cortex, *yellow* = inner parietal cortex, *red* = CA1 hippocampus, *magenta* = caudal internal capsule, *scale bar* = 100 μm

**Fig. 2:**
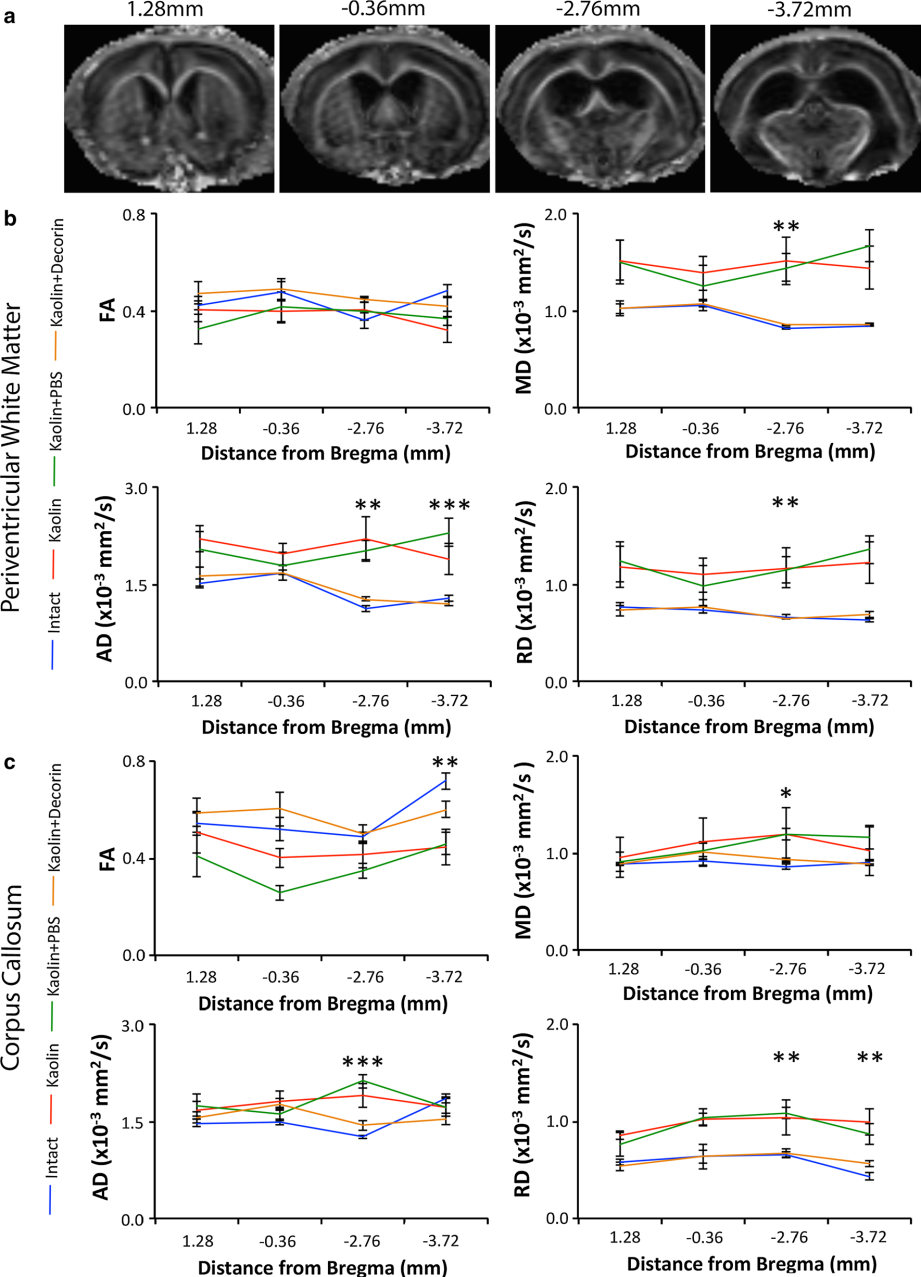
Decorin reduced hydrocephalus induced abnormalities in the caudal corpus callosum and periventricular white matter as evident from DTI. **a** Representative FA images of the locations at which the corpus callosum and periventricular white matter were analysed. *Section 1* (1.28 mm anterior to Bregma) and *Section 2* (0.36 mm posterior to Bregma) are classified as the rostral periventricular white matter and corpus callosum. *Section 3* (2.76 mm posterior to Bregma) and *Section 4* (3.72 mm posterior to Bregma) refer to the caudal periventricular white matter and corpus callosum. *Line graphs* displaying decorin’s ability to reduce abnormalities in the (**b**) corpus callosum and (**c**) periventricular white matter on DTI; *blue* = Intact, *green* = kaolin, *red* = kaolin + PBS, *orange* = kaolin + decorin [Intact (n = 4), kaolin (n = 4), kaolin + PBS (n = 6), kaolin + decorin (n = 5)]. *Error bars* represent the standard error of the mean; *p < 0.05, **p < 0.01, ***p < 0.001

Prior to commencing voxel-based analysis, double blinding was introduced to prevent group identification. Using the software, DSI Studio (DSI Studio, Pittsburgh, PA), DTI images were reconstructed and processed to produce voxel based maps, from which regions of interest (ROIs) could be analysed. The seven ROIs selected include: corpus callosum, periventricular white matter, caudal and rostral internal capsule, outer parietal cortex, inner parietal cortex and CA1 hippocampus. DTI parameter values from four serial sections (1.28 mm anterior to Bregma to 3.72 mm posterior to Bregma) of the corpus callosum and periventricular white matter were analysed in a total of 17 rats [Intact (n = 4), kaolin (n = 4), kaolin + PBS (n = 5), kaolin + decorin (n = 4)]. The rostral corpus callosum and periventricular white matter sections were defined as 1.28 and −0.36 mm from Bregma. The caudal corpus callosum and periventricular white matter sections were derived from −2.76 to −3.72 mm from Bregma (Fig. 2a). The remaining five ROIs were analysed in 16 rats, with four animals being examined in each experimental group. Three sections were independently analysed for the CA1, caudal internal capsule, outer and inner parietal cortex from 0.36 to 3.72 mm posterior to Bregma. An average of two sections from 1.28 mm anterior to Bregma to 0.36 mm posterior to Bregma were individually analysed for the rostral internal capsule. ROIs were identified using a FA voxel based map and an analogous DTI image (Fig. 1). The mean FA, MD, AD and RD values were calculated for each ROI of each animal.

### Tissue preparation for histology

Rats were euthanised and immediately perfused transcardially with PBS followed by 4 % paraformaldehyde (Alfa Aesar, Ward Hill, MA, USA) in PBS. Brains were immersed in 4 % paraformaldehyde overnight at 4 °C, cryoprotected by sequential immersion in 10, 20 and 30 % sucrose solutions in PBS at 4 °C and embedded in optimum cutting temperature embedding matrix (Fisher Scientific). Subsequent sectioning and staining of the tissue was conducted at the University if Birmingham. Coronal sections 15-µm thick were cut on a Bright cryostat (Bright Instrument, Huntingdon, UK), serially mounted and stored at −20 °C before staining.

### Antibodies

Myelin integrity was assessed with an antibody against myelin basic protein (MBP; rat, Merck Millipore, Watford, UK, MAB386). Antibodies against glial fibrillary acidic protein (GFAP; mouse, Sigma-Aldrich, G3893) and OX-42 (CD-11b; mouse, Serotec, Kidlington, UK, MCA527R) were used to assess gliosis and the extent of oedema resolution was examined by aquaporin-4 (AQP4) antibody staining (chicken, Genway, San Diego, CA, USA, 07GA0175-070718).

### Fluorescent immunohistochemistry

Immunohistochemistry was conducted on the caudal cerebrum of 19 rats [Intact (n = 4), kaolin (n = 4), kaolin + PBS (n = 6), kaolin + decorin (n = 5)]. All selected sections were at least −2.5 mm posterior to Bregma and corresponded with the location of the DTI sections. Sections were washed in PBST (10 mM PBS pH 7.4 containing 0.3 % Tween20) and blocked in 2 % bovine serum albumin (BSA) and 15 % normal goat serum in PBST at room temperature for 1 h. Subsequently, sections were washed in PBST, before being incubated at 4 °C overnight in primary antibody diluting buffer containing PBST and 2 % BSA. After washing in PBST the sections were incubated for 1 h in secondary antibody solution (Alexa Fluor^®^ 488 or 594 labelled secondary antibodies (Life Technologies, Paisley, UK) in PBST with 2 % BSA and 1.5 % normal goat serum) at room temperature, in the dark. After further PBST washes, sections were mounted in Vectashield containing DAPI (Vector Laboratories, Peterborough, UK). The Zeiss Axioplan 2 imaging epifluorescent microscope (Carl Zeiss, Germany) and the AxioCam Hrc (Carl Zeiss, Jena, Germany) were used to view and capture images under the same conditions for each antibody at ×400 magnification.

### Pixel based analysis of immunofluorescent staining

Quantitative analysis was undertaken using the software, Image J and all analyses were undertaken with the operator masked to the experimental group. Images for each immunofluorescent stain were processed identically before being analysed. For each image analysed, four randomly placed regions of interest (ROIs) were drawn with each ROI being 2.96 mm wide and 1.57 mm in height. For the corpus callosum, periventricular white matter and CA1 and CA3 hippocampal regions, a mean of 16 ROIs (four regions of interest × four coronal sections) were chosen per rat per stain. An average of 8 ROIs (four regions × two coronal sections) were selected for the internal capsule, caudate-putamen, parietal cortex and occipital cortex. All areas were analysed for GFAP, OX-42 and AQP4 staining however, as MBP is a marker of white matter integrity, only the corpus callosum, periventricular white matter and internal capsule were analysed for this antibody.

GFAP and OX-42 image processing included the conversion of images into a gray scale format prior to spatial filtering, thresholding and despeckling of the images using Image J. Images stained for AQP4 and MBP were identically processed to the GFAP and OX-42 images except thresholding was not performed. The mean percentage area of GFAP, OX-42, AQP4 and MBP positive staining, for each experimental group was calculated.

### Bright field microscopy

In order to assess hippocampal size, one cerebral section, at least 2.5 mm posterior to Bregma from each experimental animal was examined at ×10 magnification using the Nikon SM21500 dissecting microscope (Nikon, Tokyo, Japan). Images were captured with a Nikon ds-2mv high-resolution camera (Nikon). Hippocampal area was assessed by using the Image J software analyze area tool.

### Statistics

Statistical analysis was conducted using SPSS software, version 22 (IBM, Armonk, NY). Normally distributed data were analysed using a one-way ANOVA followed by a post hoc Tukey test. In the absence of normality, data were analysed using the Kruskal–Wallis test and tested for significant pairwise comparisons. Normally distributed data were expressed as the mean ± standard error of the mean (SEM). Correlation analysis was performed using a two-tailed Spearman’s correlation test. As immunohistochemistry analysis was performed on caudal sections, mean DTI data from Section 2.76 and 3.72 mm posterior to Bregma were used for correlation analysis. Correlation analysis was not undertaken for the inner parietal cortex, caudate-putamen, occipital cortex or CA3 region of the hippocampus because DTI analysis was not being performed in these areas. Values were considered statistically significant when p values were *p < 0.05, **p < 0.01, ***p < 0.001 and ****p < 0.0001.

## Results

### Decorin reduces hydrocephalus induced DTI changes in the caudal periventricular white matter and corpus callosum

In the caudal periventricular white matter (−2.76 and −3.72 mm from Bregma), compared to Intact controls (0.83 ± 0.00 and 1.20 ± 0.02, respectively; Fig. 2b), the kaolin and kaolin + PBS groups displayed a significant increase in the MD (1.48 ± 0.22, p = 0.023 and 1.56 ± 0.15, p = 0.031 respectively) and AD (2.04 ± 0.27, p = 0.020 and 2.16 ± 0.17, p = 0.018, respectively). By contrast, the MD of decorin treated rats (0.85 ± 0.00) was significantly lower in comparison to kaolin (1.48 ± 0.22, p = 0.032) and kaolin + PBS (1.56 ± 0.15, p = 0.044) rats. No significant differences were observed in any DTI parameters in the rostral periventricular white matter (1.28 and −0.36 mm from Bregma) between the four experimental groups.

In the caudal corpus callosum (−2.76 and −3.72 mm from Bregma), the AD for kaolin (1.80 ± 0.15, p = 0.010) and kaolin + PBS (1.92 ± 0.12, p = 0.001) groups were significantly higher than Intact controls (1.56 ± 0.05; Fig. 2c). Moreover, kaolin + PBS rats displayed a significant elevation in the MD (1.17 ± 0.08, p = 0.030) and RD (1.00 ± 0.06, p = 0.025), compared to Intact animals (0.88 ± 0.02 and 0.54 ± 0.02, respectively). Furthermore, decorin treatment significantly reduced the AD (1.49 ± 0.02, p = 0.004) and RD (0.62 ± 0.03, p = 0.034) compared to the kaolin + PBS animals (1.92 ± 0.12 and 1.00 ± 0.06, respectively) in the caudal corpus callosum. No significant differences existed between decorin treated rats and Intact controls for all DTI parameters in the caudal corpus callosum. Similar to the rostral periventricular white matter, in the rostral corpus callosum (1.28 and −0.36 mm from Bregma), there were no significant differences in the DTI parameters between the experimental groups.

Alongside the corpus callosum and the periventricular white matter, five other regions of interest were examined by DTI voxel based analysis (Additional file 2: Figure S2). Significant differences in the four DTI parameters were not observed in the outer or inner parietal cortex (Additional file 2: Figure S2a, b). However, the FA of the kaolin group was significantly lower (0.11 ± 0.02, p = 0.036) than Intact controls (0.16 ± 0.01) in the CA1 region of the hippocampus (Additional file 2: Figure S2c). Moreover, in the caudal internal capsule (Additional file 2: Figure S2d), decorin (0.83 ± 0.01, p = 0.003) reduced the decrease in the MD observed in kaolin + PBS animals (0.77 ± 0.00). In the rostral internal capsule (Additional file 2: Figure S2e), kaolin + PBS rats displayed significantly greater FA (0.16 ± 0.01, p = 0.022) and AD (1.70 ± 0.28, p = 0.039) compared to Intact controls (0.23 ± 0.00 and 0.68 ± 0.23, respectively).

### Ventriculomegaly is greatest in the caudal hydrocephalic cerebrum and correlates with DTI parameters

As DTI parameter abnormalities were predominantly observed in the caudal cerebrum, we investigated whether non-uniform ventriculomegaly occurs in the basal cistern model of communicating hydrocephalus. In the kaolin and kaolin + PBS groups, the ventricles expanded significantly (p < 0.05) rostrally (8.04 ± 1.74 and 10.12 ± 3.83 mm^3^, respectively) and caudally (16.22 ± 2.76 and 21.00 ± 5.43 mm^3^, respectively) compared to the Intact controls (rostral = 1.26 ± 0.11 and caudal = 0.93 ± 0.10 mm^3^). Furthermore, significant changes in the mean differences between the rostral versus caudal ventricular volume were discovered (Table 1).

**Table 1:** Significant changes in the mean differences between the rostral versus caudal ventricular volumes amongst the four experimental groups

	p
Intact vs Kaolin	0.005
Intact vs Kaolin + PBS	<0.001
Intact vs Kaolin + decorin	0.946
Kaolin vs Kaolin + PBS	0.530
Kaolin vs Kaolin + decorin	0.015
Kaolin + PBS vs Kaolin + decorin	0.001

Rostrally, ventricular volume significantly correlated with the FA, MD, AD and RD measurements of the corpus callosum and periventricular white matter (Table 2). Likewise, the caudal ventricular volume correlated with all DTI parameter measures in the corpus callosum and all except the AD in the periventricular white matter (Table 2).

**Table 2:** DTI parameter values of the corpus callosum and periventricular white matter correlated with rostral and caudal ventricular volume

DTI parameter	R (Spearman’s rho)	p
*Rostral ventricular volume*		
CC FA	0.831	<0.001
CC MD	0.539	0.026
CC AD	0.527	0.030
CC RD	0.733	0.001
PVWM FA	0.949	<0.001
PVWM MD	0.706	0.002
PVWM AD	0.507	0.038
PVWM RD	0.642	0.005
*Caudal ventricular volume*		
CC FA	−0.676	0.003
CC MD	0.723	0.001
CC AD	0.777	<0.001
CC RD	0.838	<0.001
PVWM FA	−0.520	0.033
PVWM MD	0.537	0.026
PVWM AD	0.441	0.076
PVWM RD	0.547	0.023

Statistically significant correlations = p < 0.05

*CC* corpus callosum, *PVWM* periventricular white matter, *FA* fractional anisotropy, *MD* mean diffusivity, *AD* axial diffusivity, *RD* radial diffusivity, *R* correlation coefficient (Spearman’s rho)

### Decorin reduces caudal periventricular white matter cytopathology

**Fig. 3:**
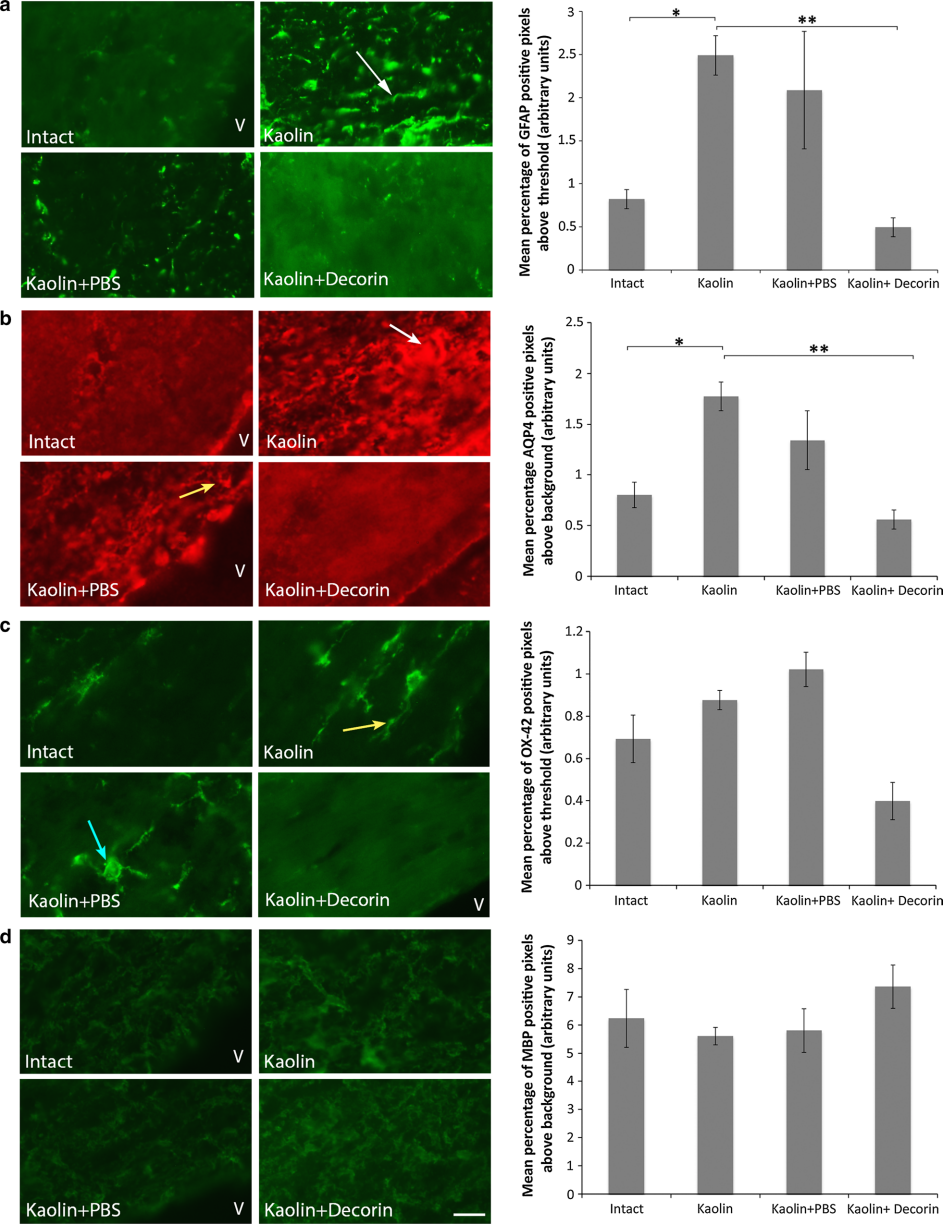
Decorin prevented an increase in GFAP and AQP4 in the periventricular white matter. Representative images comparing the level of (**a**) GFAP immunostaining (*green*), (**b**) AQP4 immunostaining (*red*), (**c**) OX-42 immunostaining (*green*) and (**d**) MBP immunostaining (*green*) in the periventricular white matter; *scale bar* = 10 μm. **a** kaolin and kaolin + PBS rats displayed thickening of astrocytic processes (*white arrow*). **b** Accumulation of AQP4 staining was observed in kaolin rats (*white arrow*). AQP4 was further arranged around the circumference of blood vessels (*yellow arrow*). **c** Elongated, amoeboid microglia (*yellow arrow*) were particularly evident in kaolin rats. Microglia of kaolin + PBS rats were captured transitioning from branched resting microglia to activated amoeboid microglia (*blue arrow*). **d** Decorin treatment improved the myelin loss and disorganisation present in kaolin and kaolin + PBS rats (*white arrow*). Each corresponding bar graph displays the mean percentage of GFAP, AQP4, OX-42 or MBP positive pixels above threshold or background in the periventricular white matter across the four experimental groups; *V* lateral ventricle, *error bars* represent the standard error of the mean, *p < 0.05, **p < 0.01

As DTI parameter abnormalities were observed in the caudal periventricular white matter, corresponding immunohistochemistry analysis was conducted to aid hydrocephalic cytopathology characterisation of these tissues. We determined that the levels of GFAP positive immunostaining (Fig. 3a) were significantly increased in kaolin rats (2.49 ± 0.23 %, p = 0.048) compared to Intact controls (0.82 ± 0.11 %) indicating the presence of astrogliosis in the caudal periventricular white matter. Furthermore, the GFAP positive astrocytes of hydrocephalic animals exhibited features typical of reactive astrocytic morphology; cytoplasmic processes underwent thickening in kaolin and kaolin + PBS rats. The increase in GFAP positive staining observed in the kaolin rats (2.49 ± 0.23 %) was prevented with decorin treatment (0.49 ± 0.11 %, p = 0.002). Additionally, kaolin (1.77 ± 0.14 %, p = 0.040) and kaolin + PBS rats (1.34 ± 0.29 %, p = 0.056) displayed a significant and non-significant increase respectively in periventricular white matter AQP4 positive immunostaining (Fig. 3b) compared to Intact controls (0.80 ± 0.12 %). Importantly, AQP4 immunostaining in kaolin + decorin treated rats was significantly lower (0.56 ± 0.09 %, p = 0.006) than in kaolin animals (1.77 ± 0.14 %). No significant difference existed in periventricular white matter AQP4 immunostaining between decorin treated and Intact control rats (p = 0.860). In contrast, significant differences in OX-42 and MBP levels were not present between the experimental groups in the periventricular white matter (Fig. 3c, d). Furthermore, significant differences in GFAP, OX-42, MBP and AQP4 immunostaining were not present between the experimental groups in the caudal corpus callosum (Additional file 3: Table S1).

### Decorin protects from myelin damage in the caudal internal capsule

**Fig. 4:**
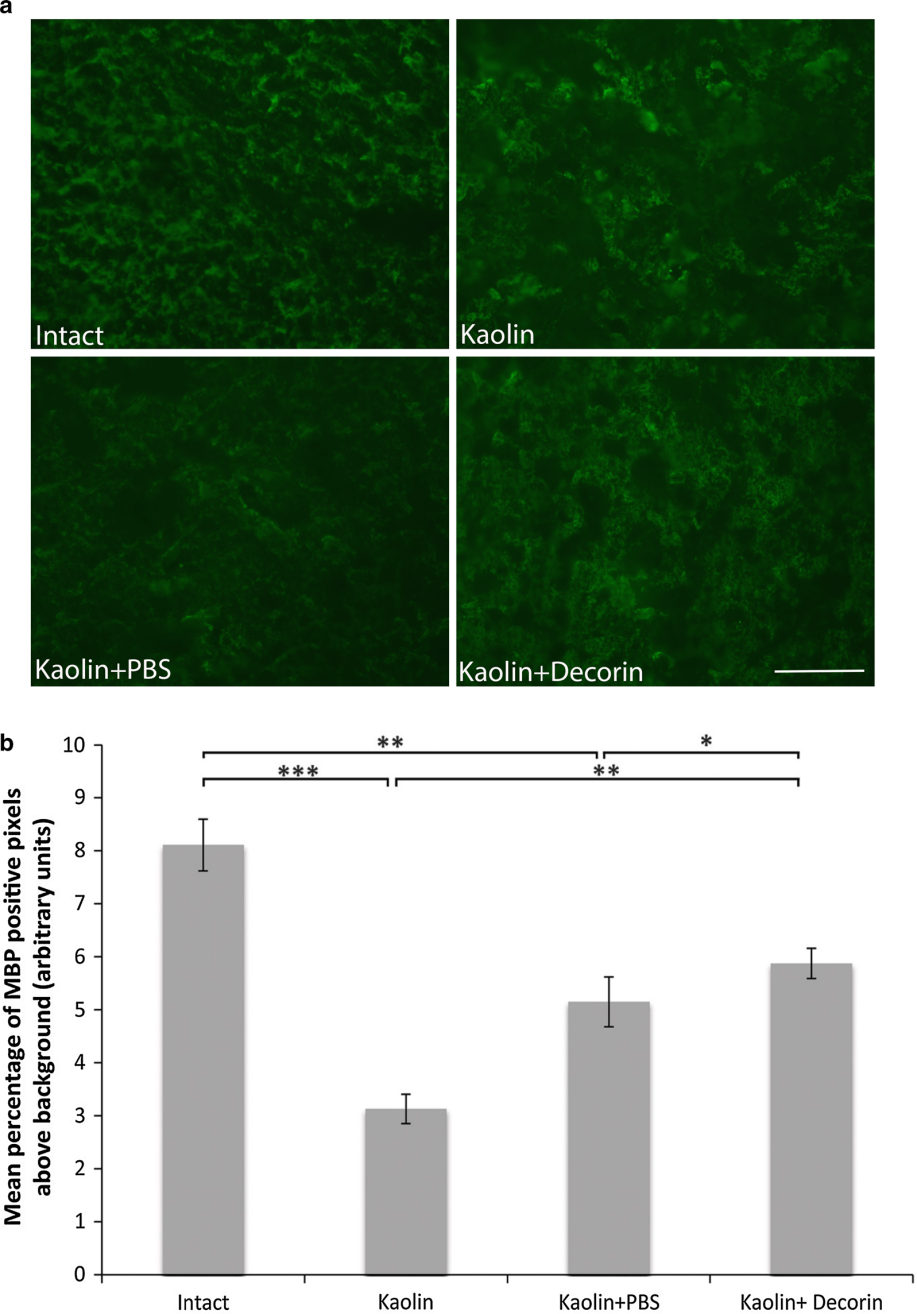
Decorin prevented myelin loss in the caudal internal capsule. **a** Representative images comparing caudal internal capsule MBP immunostaining (*green*) across the four experimental groups. Myelin organisation was better maintained with decorin use. **b** A* bar graph* displaying the mean percentage of MBP positive pixels above threshold in the internal capsule across the four experimental groups. Decreased MBP levels were present in kaolin and kaolin + PBS rats which was incompletely attenuated with decorin treatment; *error bars* represent the standard error of the mean, *p < 0.05, **p < 0.01, ***p < 0.001; *scale bar* = 50 μm

Although significant differences in myelin levels were not present in the caudal corpus callosum and caudal periventricular white matter, loss of myelin (assessed by measurement of MBP) in the caudal internal capsule was present in kaolin and kaolin + PBS animals (Fig. 4); compared to Intact controls (8.11 ± 0.49 %), a decrease in MBP immunostaining was present in kaolin (3.13 ± 0.28 %, p < 0.001) and kaolin + PBS (5.15 ± 0.47 %, p = 0.001) rats. Furthermore, decorin treated rats displayed higher MBP levels (5.87 ± 0.29 %) compared to kaolin (3.13 ± 0.28 %, p = 0.002) and kaolin + PBS rats (5.15 ± 0.47 %, p = 0.018), although the levels did not quite reach the same as in intact rats (p = 0.009), indicating some myelin protection. Qualitatively, the longitudinal organisation of myelin was disrupted, with discontinuity present along the length of the myelin fibres in animals receiving kaolin and kaolin + PBS compared to Intact controls. The regular parallel arrangement of MBP staining was protected with decorin treatment.

### Decorin attenuates hippocampal atrophy in communicating hydrocephalus

Upon examining total hippocampal area, significant differences were present between the four experimental groups; a decrease in normalised hippocampal area was identified in the kaolin (47 ± 9 %, p = 0.006) and kaolin + PBS rats (69 ± 9 %, p < 0.001) compared to Intact controls (100 ± 6 %). Decorin treatment attenuated the hippocampal atrophy (89 ± 7 %, p = 0.008) compared to kaolin rats but failed to maintain the hippocampal size to that in Intact controls (100 ± 6 %, p < 0.001). In the CA1 region of the hippocampus, similar levels of GFAP, OX-42 and AQP4 were observed between all the experimental groups. In the CA3 region, GFAP levels were comparable among all four groups however kaolin rats (0.43 ± 0.02 %, p = 0.057) demonstrated a trend towards reduced OX-42 levels compared to Intact controls (0.89 ± 0.03 %), and kaolin + PBS rats (1.04 ± 0.13 %, p = 0.043) displayed a significant increase in AQP4 levels compared to Intact controls (1.60 ± 0.10 %; Additional file 4: Table S2).

### GFAP and AQP4 levels correlate significantly in the corpus callosum, periventricular white matter, caudate-putamen, parietal cortex and occipital cortex

No significant differences in AQP4, OX-42 and GFAP immunostaining were present between the experimental groups in the caudate putamen, parietal cortex and occipital cortex (Additional file 4: Table S2). However, in the corpus callosum, periventricular white matter, caudate-putamen, parietal cortex and occipital cortex, GFAP immunostaining positively correlated with AQP4 levels (Table 3). No significant correlations were present between OX-42 and AQP4 levels in any of the regions of interest.

**Table 3:** AQP4 levels correlated significantly with the marker of gliosis, GFAP, in the corpus callosum, periventricular white matter, caudate-putamen and parietal and occipital cortex

Region of interest	R	p
Corpus callosum	0.614	0.005
Periventricular white matter	0.854	<0.001
CA1 hippocampus	0.332	0.166
CA3 hippocampus	0.446	0.056
Internal capsule	0.291	0.226
Caudate-putamen	0.495	0.043
Parietal cortex	0.528	0.020
Occipital cortex	0.607	0.006

Statistically significant correlations = p < 0.05

*R* correlation coefficient

### Hydrocephalic cytopathology correlates with abnormalities on DTI

In the caudal corpus callosum, increased astrocyte (GFAP) and AQP4 levels positively correlated with the AD (Table 4). Furthermore, in the caudal periventricular white matter (Table 4), GFAP and AQP4 positively correlated with AD, MD and RD. Moreover, the presence of cytopathology discouraged anisotropic water diffusion in the caudal periventricular white matter as the FA negatively correlated with astrocyte (GFAP), microglial (OX-42) and AQP4 immunostaining. A negative correlation was also present between myelin levels (MBP) and the MD of the caudal periventricular white matter.

**Table 4:** The marker of gliosis, GFAP, and AQP4 levels correlated with DTI parameter values in the periventricular white matter

ROI	Immunostain	DTI parameter	R	p
Corpus callosum	GFAP	FA	−0.370	0.144
		MD	0.306	0.232
		AD	0.600	0.011*
		RD	0.424	0.090*
	OX-42	FA	−0.086	0.743
		MD	0.002	0.993
		AD	0.352	0.165
		RD	0.120	0.646
	AQP4	FA	−0.323	0.205
		MD	0.191	0.462
		AD	0.566	0.018*
		RD	0.409	0.103
	MBP	FA	0.091	0.729
		MD	−0.031	0.903
		AD	0.159	0.541
		RD	−0.115	0.660
Periventricular white matter	GFAP	FA	−0.485	0.048*
		MD	0.647	0.005*
		AD	0.667	0.003*
		RD	0.680	0.003*
	OX-42	FA	−0.495	0.043*
		MD	0.292	0.256
		AD	0.299	0.244
		RD	0.213	0.411
	AQP4	FA	−0.640	0.006*
		MD	0.799	<0.001*
		AD	0.801	<0.001*
		RD	0.829	<0.001*
	MBP	FA	0.346	0.174
		MD	−0.495	0.043*
		AD	−0.360	0.155
		RD	−0.458	0.064*

*FA* fractional anisotropy, *MD* mean diffusivity, *AD* axial diffusivity, *RD* radial diffusivity, *R* the correlation coefficient

* Statistically significant correlations = p < 0.05

## Discussion

This study demonstrates that decorin is able to protect and maintain DTI parameter values at normality in the caudal corpus callosum and caudal periventricular white matter. Likewise, decorin prevents astrogliosis and oedema in the caudal periventricular white matter and preserves myelin integrity in the caudal internal capsule. Furthermore, cytopathology in communicating hydrocephalus is predominantly localised to the caudal cerebrum. Moreover, DTI parameters correlate with cytopathology specifically in the caudal periventricular white matter. DTI is therefore a useful tool to act as a surrogate measure of cytopathology in communicating hydrocephalus.

Recent studies in post-haemorrhagic hydrocephalus suggest that occipital horn enlargement is greater and precedes the dilation of the remaining ventricular system [46–48]. This asymmetry is a pattern that is repeated in other types of hydrocephalus including congenital hydrocephalus [49] and idiopathic chronic hydrocephalus [50], although this feature has not been explored in depth or quantitatively. In feline infants [51], neonatal rats [41, 52] and adult dogs [53] with non-communicating hydrocephalus induced by kaolin injections into the cisterna magna, the occipital horns of the lateral ventricles are conspicuously larger than the frontal horns. Our results support these findings, albeit in an experimental model of communicating hydrocephalus, by showing that caudal portions of the lateral ventricles expand more than frontal regions, and DTI abnormalities are largely situated in the caudal white matter.

Asaaf and colleagues [54] suggested that DTI could be used as a marker of white tissue compression in obstructive hydrocephalus. Furthermore there have been no observed DTI changes in the white matter of idiopathic intracranial hypertension patients (high ICP but no ventriculomegaly [55]) suggesting that compression of tissue impacts DTI parameters. In the caudal periventricular white matter, our findings replicate the abnormalities in the MD, AD and RD observed in hydrocephalic children [29–31]. In contrast to our findings, the MD of the periventricular white matter does not increase in post-haemorrhagic hydrocephalus in adults [56], therefore the maturity of the brain appears to influence DTI alterations. Similar to the findings of Yuan et al. [41] in rats of the same age with obstructive hydrocephalus (blockage of the cisterna magna), our communicating hydrocephalic animals display an increase in MD and reduced FA in the caudal corpus callosum. Our study has also revealed an increase in the AD and RD of the caudal corpus callosum in communicating hydrocephalus, which is additionally preventable by decorin treatment.

The cytopathology observed in our model supports current literature and is largely preventable with decorin treatment [10, 12, 57–64]. Although white matter abnormalities discovered were similar to those in hydrocephalic children [29–34], decorin was only able to protect the internal capsule from myelin damage. TGF-β mediated signaling promotes central nervous system myelination by enhancing oligodendrocyte progenitor cell differentiation and maturation [65, 66]. It is possible that internal capsule oligodendrocyte progenitor cells may be more susceptible to abnormalities in TGF-β signaling than those of the corpus callosum or periventricular white matter, hence explaining the observed result.

The relationship between DTI parameters and cerebral histopathological changes has been discussed extensively in recent literature [27, 31, 32, 67–71]. Events that discourage directional water movement, such as interstitial oedema and neurodegeneration cause a decline in the FA [27, 28, 72–77]. The AD and RD are two DTI parameters that influence the FA and provide insight into axonal and myelin integrity, respectively [73, 76]. Both the AD and RD are also influenced by gliotic tissue changes [27, 72, 76]. Furthermore, an increase in average amount of diffusion in a given volume of tissue, caused by the presence of interstitial oedema or the loss of cellular barriers, results in a rise in the MD [27, 77, 78].

Consistent with the results of Yuan et al. [41] in juvenile rats with obstructive hydrocephalus, our findings in communicating hydrocephalus show positive correlations between GFAP increases and MD, AD, and RD in caudal periventricular white matter. Likewise, the increased levels of OX-42 (a marker of microglia) correlated negatively with the FA. This result may seem surprising since cytoarchitecturally in the periventricular white matter of kaolin and kaolin + PBS rats, the majority of microglial processes were longitudinally oriented; therefore an increase in FA would have been predicted [58]. However, as others have reported in congenital hydrocephalus [64], the cell bodies of reactive microglia in the periventricular white matter of our kaolin and kaolin + PBS animals were enlarged and widened. This cytopathological characteristic may have obstructed the parallel diffusion of water causing the FA to decrease. Since the pathophysiology of hydrocephalus is extremely multifactorial, it is unlikely that glial alterations alone exert a causative effect on DTI parameters.

The MBP levels of the caudal periventricular white matter correlate with the MD. These results corroborate the current literature; by increasing the volume of the extracellular space, myelin disorganisation and demyelination increases the MD of water molecules [27, 73, 76]. In support of the Tourdias et al. [78] report on communicating hydrocephalus, AQP4 levels positively correlated with the MD measurements in the caudal periventricular white matter. AQP4 levels also positively correlated with the FA, RD and more interestingly the AD measurements. Although sparse literature exists on the relationship between AQP4 and AD, we suggest that the removal of excess interstitial fluid by high levels of AQP4 may promote the unobstructed parallel movement of water through the periventricular white matter, hence resulting in an increase in the AD measurement. Further investigation of this hypothesis needs to be undertaken in order to substantiate such claim.

Here we have used kaolin to induce communicating hydrocephalus to help us determine the therapeutic effects of decorin. It is important to recognize the possibility that some decorin-treated animals may not have developed ventriculomegaly simply because of induction failures. However, it is unlikely that a significant proportion of the decorin-treatment group would not develop ventriculomegaly given the fact that 82 % of kaolin-only or kaolin + PBS animals demonstrated significantly enlarged ventricles [12]. In addition, 79 % of adult rats with identical induction procedures developed ventriculomegaly [79]. Thus, we believe that the improvements in the decorin-treated animals were due primarily to the drug intervention. Another consideration of the study is that the kaolin model of hydrocephalus is not the most clinically relevant model, however it is the best characterised and most widely used, successfully replicating the development and pathophysiological consequence of acquired hydrocephalus. Kaolin induces an inflammatory response with concomitant deposition of fibrosis in areas of the subarachnoid space close to the injection site [80, 81] which is very similar to that observed in subarachnoid haemorrhage rat models [82]. The next step would be to determine the effects of decorin in a post haemorrhagic model. Recently, Yan et al. [83] demonstrated that pretreating rats with decorin in a subarachnoid haemorrhage model led to a reduction in ventriculomegaly and markers of fibrosis, indicating that decorin may have beneficial effects in subarachnoid haemorrhage. However further work needs to be conducted looking at the changes in cerebral cytopathology and microstructure with decorin treatment in this model.

## Conclusions

Our findings highlight the therapeutic potential of decorin to attenuate hydrocephalus-induced changes in astrogliosis, oedema and demyelination, particularly in the caudal periventricular white matter. Our study also helps to validate the use of DTI as a surrogate marker of cytopathology in communicating hydrocephalus and demonstrates that the caudal region of the brain appears to be the most affected, showing the greatest changes in ventriculomegaly, DTI and cytopathological measures in our experimental model.

## Appendix 1

Diffusion tensor imaging (DTI). DTI is a specialised magnetic resonance imaging technique that is used to gain greater appreciation of white matter disease-related pathophysiology via probing the random translational motion of water molecules [26]. The scalar parameters in DTI provide a quantitative method to assess cerebral water motion by specifically examining the magnitude and direction of water diffusion, which is quantified by measuring key parameters such as axial diffusivity (AD), radial diffusivity (RD) and their derivatives mean diffusivity (MD) or fractional anisotropy (FA). The FA values provide insight into the anisotropy of water diffusion. Water may diffuse isotropically, i.e. equally in all directions, or along a specific direction, therefore becoming anisotropic in nature. Moreover, the FA can be influenced by changes in microstructural integrity [27]; neurodegeneration and axonal reorganization hinders isotropic water movement, decreasing the FA. Furthermore, in ventriculomegaly-induced cerebral compression, increased AQP4 levels and gliosis raise the FA [74]. The RD and AD are two parameters that directly influence the FA. In white matter, the proportion of water diffusing perpendicular to neuronal fibres is assessed by RD, whilst the degree of water diffusion parallel to tract orientation is determined by the AD. Increased RD and AD are indicators of myelin and axonal integrity, respectively. Both RD and AD are also reported to increase upon astrogliosis [27]. Quantification of the average magnitude of diffusion in a given volume of tissue is provided by the MD value. MD is decreased by the presence of cellular barriers [80]. In contrast, interstitial edema and greater AQP4 and microglial presence are responsible for a rise in the MD [80].

## Additional files

10.1186/s12987-016-0033-2 Decorin prevents ventriculomegaly assessed by T2 weighted MRI images. A bar graph showing a significant increase in ventricular volume in hydrocephalic rats compared to Intact controls. Furthermore, ventriculomegaly was prevented with 2.5 μg/0.5 µl/h infusion of human recombinant decorin treatment; ***p < 0.001. Corresponding representative T2- weighted MRI images displaying the differences in ventricular volume between the experimental groups in the rostral and caudal brain. Arrows highlight the size of the lateral ventricles (LV) or third ventricle (3V). Ventriculomegaly was evident by MRI in the hydrocephalic rats but not in Intact controls or rats that received decorin. Adapted from [12] with permission.
10.1186/s12987-016-0033-2 DTI parameter abnormalities in the CA1 hippocampus, rostral and caudal internal capsule. Mean values + the standard error of the means are displayed for the fractional anisotropy (FA), mean diffusivity (MD), axial diffusivity (AD) and radial diffusivity (RD) in the (A) outer parietal cortex, (B) inner parietal cortex, (C) CA1 hippocampus, (D) caudal internal capsule and (E) rostral internal capsule; significant differences (p < 0.05) in the DTI parameter values from intact (*) or kaolin + PBS (+) levels are presented.
10.1186/s12987-016-0033-2 In the corpus callosum, no significant cytopathological changes were observed in hydrocephalic animals. The mean values ± the standard error of the means of GFAP, OX-42, AQP4 and MBP immunostaining in the four different experimental groups are expressed.
10.1186/s12987-016-0033-2 AQP4, GFAP and OX-42 levels in the CA1 and CA3 hippocampus, internal capsule, caudate-putamen, parietal cortex and occipital cortex in the four experimental groups; values represent the mean ± standard error of the means, * = p<0.05.

## Abbreviations

AD: axial diffusivity
AQP4: aquaporin-4
CC: corpus callosum
GFAP: glial acidic fibrillary protein
DTI: diffusion tensor imaging
MBP: myelin basic protein
MD: mean diffusivity
OX-42: part of the C3 complement receptor
PVWM: periventricular white matter
ROI: region of interest
RD: radial diffusivity
TGF-β: transforming growth factor-β
