# Supplementary figures and images for: Diffusion tensor imaging with direct cytopathological validation: characterisation of decorin treatment in experimental juvenile communicating hydrocephalus
Source: Fluids Barriers CNS. 2016 May 31;13:9. doi: 10.1186/s12987-016-0033-2 (PMC4888658; doi:10.1186/s12987-016-0033-2)

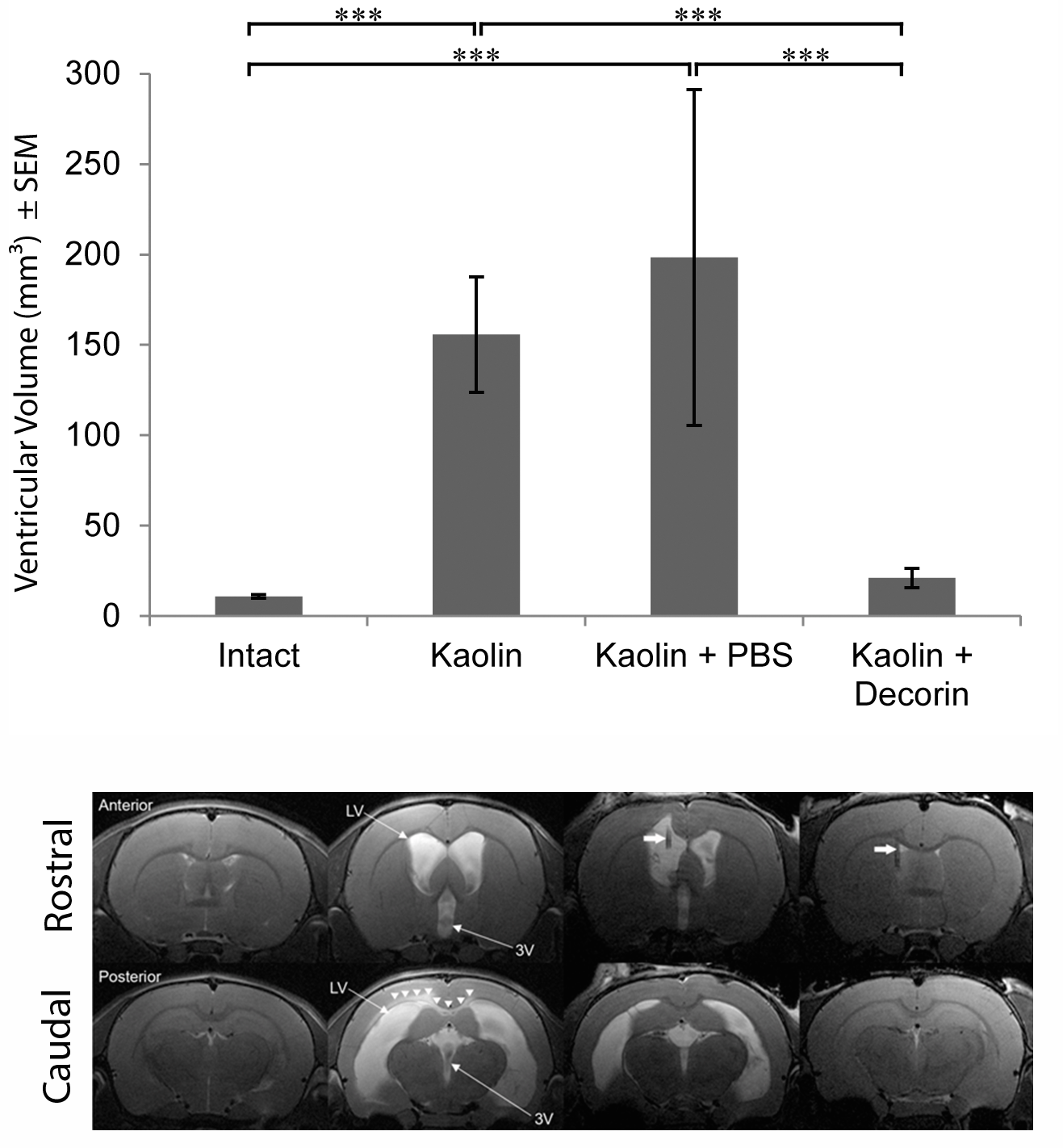

Supplement: Supplementary file 1 — 10.1186/s12987-016-0033-2 Decorin prevents ventriculomegaly assessed by T2 weighted MRI images. A bar graph showing a significant increase in ventricular volume in hydrocephalic rats compared to Intact controls. Furthermore, ventriculomegaly was prevented with 2.5 μg/0.5 µl/h infusion of human recombinant decorin treatment; ***p < 0.001. Corresponding representative T2- weighted MRI images displaying the differences in ventricular volume between the experimental groups in the rostral and caudal brain. Arrows highlight the size of the lateral ventricles (LV) or third ventricle (3V). Ventriculomegaly was evident by MRI in the hydrocephalic rats but not in Intact controls or rats that received decorin. Adapted from [12] with permission. [file 12987_2016_33_MOESM1_ESM.docx]

**
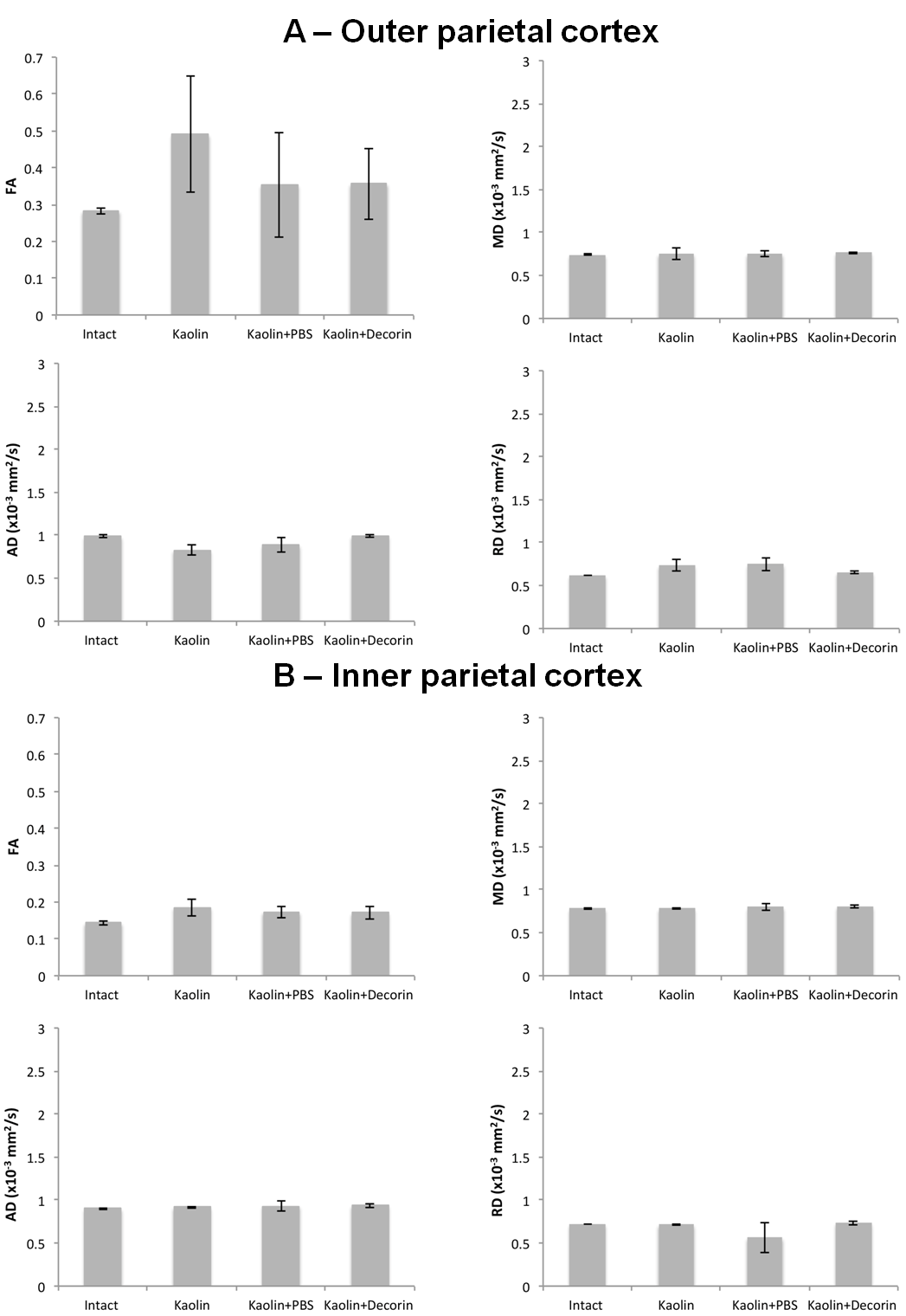
**


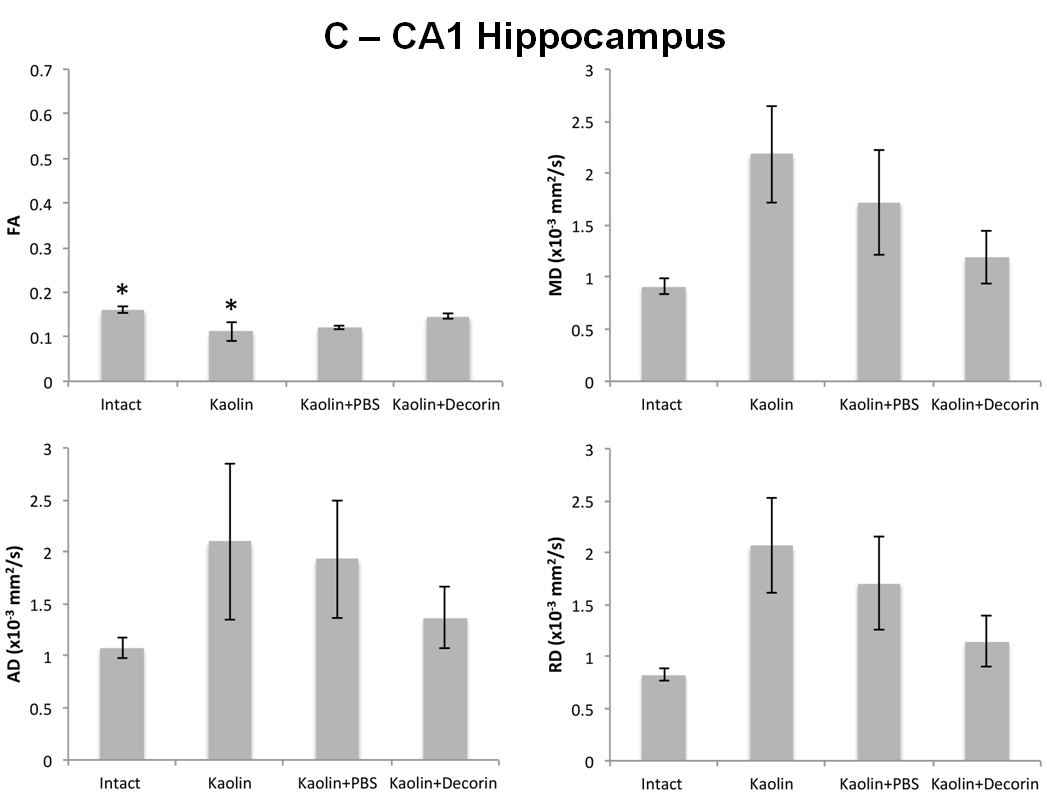


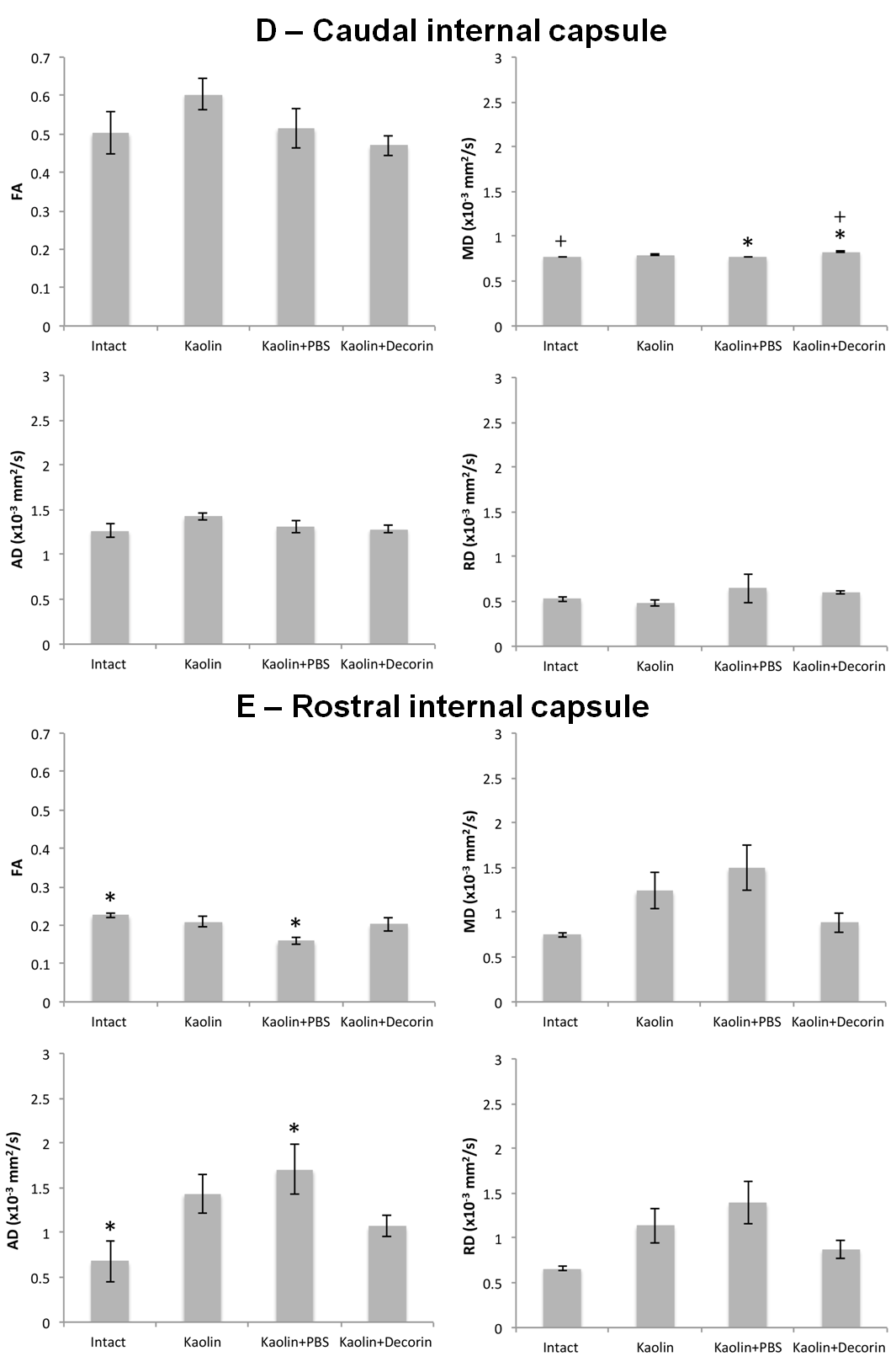

Supplement: Supplementary file 2 — 10.1186/s12987-016-0033-2 DTI parameter abnormalities in the CA1 hippocampus, rostral and caudal internal capsule. Mean values + the standard error of the means are displayed for the fractional anisotropy (FA), mean diffusivity (MD), axial diffusivity (AD) and radial diffusivity (RD) in the (A) outer parietal cortex, (B) inner parietal cortex, (C) CA1 hippocampus, (D) caudal internal capsule and (E) rostral internal capsule; significant differences (p < 0.05) in the DTI parameter values from intact (*) or kaolin + PBS (+) levels are presented. [file 12987_2016_33_MOESM2_ESM.docx]
