# Supplementary material for: Diffusion tensor imaging with direct cytopathological validation: characterisation of decorin treatment in experimental juvenile communicating hydrocephalus
Source: Fluids Barriers CNS. 2016 May 31;13:9. doi: 10.1186/s12987-016-0033-2 (PMC4888658; doi:10.1186/s12987-016-0033-2)
Supplement: Supplementary file 3 — 10.1186/s12987-016-0033-2 In the corpus callosum, no significant cytopathological changes were observed in hydrocephalic animals. The mean values ± the standard error of the means of GFAP, OX-42, AQP4 and MBP immunostaining in the four different experimental groups are expressed. [file 12987_2016_33_MOESM3_ESM.docx]

**Supplementary Table 1:** In the corpus callosum, no significant cytopathological changes were observed in hydrocephalic animals. The mean values ±the standard error of the means of GFAP, OX-42, AQP4 and MBP immunostaining in the four different experimental groups are expressed.*1 column fitting figure*

|  | Intact | Kaolin | Kaolin+PBS | Kaolin+Decorin |
| --- | --- | --- | --- | --- |
| GFAP | 1.39+0.37 | 1.26+0.33 | 1.77+0.49 | 0.77+0.31 |
| OX42 | 0.72+0.12 | 0.81+0.32 | 0.89+0.38 | 0.44+0.10 |
| AQP4 | 1.48+0.14 | 1.79+0.16 | 1.44+0.10 | 1.31+0.16 |
| MBP | 7.94+1.11 | 7.00+0.10 | 6.92+0.52 | 6.94+0.40 |
