# Supplementary material for: Diffusion tensor imaging with direct cytopathological validation: characterisation of decorin treatment in experimental juvenile communicating hydrocephalus
Source: Fluids Barriers CNS. 2016 May 31;13:9. doi: 10.1186/s12987-016-0033-2 (PMC4888658; doi:10.1186/s12987-016-0033-2)
Supplement: Supplementary file 4 — 10.1186/s12987-016-0033-2 AQP4, GFAP and OX-42 levels in the CA1 and CA3 hippocampus, internal capsule, caudate-putamen, parietal cortex and occipital cortex in the four experimental groups; values represent the mean ± standard error of the means, * = p<0.05. [file 12987_2016_33_MOESM4_ESM.docx]

**Supplementary Table 2:** AQP4, GFAP and OX-42 levels in the CA1 and CA3 hippocampus, internal capsule, caudate-putamen, parietal cortex and occipital cortex in the four experimental groups; values represent the mean ± standard error of the means, *=p<0.05

*2 column fitting figure*

|  |  | Intact | Kaolin | Kaolin+PBS | Kaolin+Decorin |
| --- | --- | --- | --- | --- | --- |
| AQP4 | CA1 hippocampus | 1.48+0.14 | 1.79+0.16 | 1.44+0.10 | 1.31+0.16 |
|  | CA3 hippocampus | 1.60+0.10* | 1.44+0.18 | 1.04+0.13* | 1.24+0.11 |
|  | Internal Capsule | 0.82+0.23 | 0.70+0.25 | 0.76+0.14 | 0.77+0.23 |
|  | Caudate-putamen | 1.17+0.20 | 1.17+0.35 | 0.80+0.12 | 0.80+0.23 |
|  | Parietal cortex | 2.50+0.31 | 2.20+0.21 | 1.77+0.23 | 1.65+0.14 |
|  | Occipital cortex | 2.31+0.32 | 2.18+0.22 | 2.03+0.39 | 1.56+0.32 |
|  |  |  |  |  |  |
| GFAP | CA1 hippocampus | 1.44+0.23 | 1.31+0.32 | 1.54+0.30 | 0.93+0.25 |
|  | CA3 hippocampus | 1.79+0.15 | 1.61+0.42 | 1.70+0.31 | 1.14+0.29 |
|  | Internal Capsule | 0.45+0.15 | 0.48+0.27 | 0.76+0.40 | 0.28+0.13 |
|  | Caudate-putamen | 0.74+0.16 | 0.45+0.19 | 0.28+0.13 | 0.41+0.22 |
|  | Parietal cortex | 0.76+0.16 | 0.46+0.12 | 1.37+0.89 | 0.17+0.07 |
|  | Occipital cortex | 1.05+0.34 | 0.86+0.16 | 1.18+0.46 | 0.40+0.15 |
|  |  |  |  |  |  |
| OX-42 | CA1 hippocampus | 0.67+0.11 | 0.35+0.05 | 0.48+0.13 | 0.41+0.10 |
|  | CA3 hippocampus | 0.89+0.03 | 0.43+0.02 | 0.59+0.13 | 0.43+0.14 |
|  | Internal Capsule | 0.50+0.16 | 0.45+0.05 | 0.30+0.06 | 0.33+0.05 |
|  | Caudate-putamen | 0.73+0.16 | 0.33+0.06 | 0.55+0.16 | 0.53+0.12 |
|  | Parietal cortex | 0.33+0.04 | 0.42+0.09 | 0.28+0.06 | 0.26+0.06 |
|  | Occipital cortex | 0.43+0.11 | 0.29+0.06 | 0.52+0.19 | 0.28+0.05 |
